# Carrelizumab combined with anlotinib in the treatment of extensive-stage small cell lung cancer: A case report

**DOI:** 10.1097/MD.0000000000027138

**Published:** 2021-09-03

**Authors:** Liang Liu, Xin Zhang, Lihua Zhou, Tao Yang, Yun Qiao, Xiaodong Jiang

**Affiliations:** a Lianyungang Clinical College of Nanjing Medical University/ The First People's Hospital of Lianyungang, Lianyungang, Jiangsu, China; b Department of Oncology, The First People's Hospital of Lianyungang, Lianyungang, Jiangsu, China.

**Keywords:** anlotinib, antiangiogenesis, camrelizumab, extensive-stage small cell lung cancer, immune checkpoint inhibitor, programmed death 1

## Abstract

**Rationale:**

The emergence of immune checkpoint inhibitors has brought new breakthroughs in the treatment of small cell lung cancer (SCLC). Programmed cell death-ligand 1 inhibitors combined with chemotherapy have been approved for the first-line treatment of extensive-stage small cell lung cancer (ES-SCLC). However, programmed death 1 inhibitors have limited efficacy in the treatment of SCLC. The reason may be related to the abnormal vascular state in the tumor microenvironment.

**Patient concerns:**

A 55-year-old male patient, presenting cough and sputum for 1 month.

**Diagnoses:**

The patient was clinically diagnosed with SCLC and staged as ES-SCLC.

**Interventions:**

Etoposide combined with lobaplatin treatment every 3 weeks for 4 cycles, evaluate as progressive disease. On the basis of the original plan, combined with camrelizumab for 2 cycles, evaluation as progressive disease. Then, the patient was treated with intravenous infusion of camrelizumab plus oral anlotinib. After 4 cycles, evaluation as partial response. Then we continued to use camrelizumab combined with anlotinib treatment for the patient. At the end of 26 cycles, the chest computed tomography examination revealed that the patient had achieved complete remission.

**Outcomes:**

After treated with carrelizumab combined with anlotinib for 26 cycles, the curative effect was evaluated as complete remission, progression-free survival was 24 months and there was no immune-related adverse reaction during treatment period. Besides, the patient developed complicated hand–foot syndrome, but this symptom was significantly relieved after reducing the dosage of anlotinib.

**Lessons:**

In this case, antiangiogenesis combined with programmed death 1 inhibitors significantly inhibited tumor progression. It also indicated that anlotinib concurrent carrelizumab may be a superior choice for ES-SCLC. Further clinical trials required to confifirm its effificacy and safety.

Small cell lung cancer (SCLC) is a refractory tumor clinically due to its high degree of malignancy, sensitivity to chemotherapy, and easy recurrence. Fortunately, the emergence of immune checkpoint inhibitors (eg, cytotoxic T-lymphocyte-associated protein-4 [CTLA-4], programmed death 1 [PD-1]/ programmed cell death-ligand 1 [PD-L1], lymphocyte-activation-gene-3 [LAG-3], T cell immunoglobulin and mucin protein-3 [TIM-3]) has brought breakthroughs in the treatment of SCLC in recent years. PD-L1 inhibitors combined with chemotherapy have been approved as the first-line treatment for extensive-stage small cell lung cancer (ES-SCLC). However, PD-1 inhibitors have limited efficacy for the treatment of SCLC. The reason may be the abnormal vascular state in the tumor microenvironment. The curative effect was evaluated as a partial response, progression-free survival was 23 months and there was no immune-related adverse reaction during the treatment period. Besides, the patient developed complicated hand–foot syndrome, but was significantly relieved after reducing the dosage of anlotinib. The success of this case reveals the potential of antiangiogenesis combined with PD-1 inhibitors to improve the curative effect of treating ES-SCLC, which will provide a reference for further clinical research.

## 1 Introduction

Lung cancer is the malignant tumor with the highest morbidity and mortality in China. Among lung cancers, SCLC accounts for 15% to 20%, and about 70% of patients are first diagnosed with ES-SCLC.^[1,2]^ Clinically, ES-SCLC patients usually adopt a comprehensive treatment model, including chemotherapy, radiotherapy and antiangiogenesis therapy.^[3]^ Although the initial treatment of ES-SCLC is effective, but the prognosis of patients is poor, and the 5-year survival rate is less than 8%.^[4]^ Hence, a more effective ES-SCLC treatment plan is urgently needed clinically.

In recent years, immunotherapy has also made breakthrough progress for treating lung cancer as a new antitumor treatment modality.^[3]^ PD-1 inhibitors as a representative drug of immune checkpoint inhibitors (ICIs) have been widely used in clinical practice. Nevertheless, this treatment modality still has many shortcomings, such as individual differences, poor efficacy of single-drug therapy, and side effects caused by combined chemotherapy.^[5]^ Several clinical studies have approved that antiangiogenesis combined with PD-1 inhibitors can overall survival, progression-free survival (PFS), and objective response rate in patients with advanced nonSCLC.^[6]^ However, PD-1 inhibitors showed limited efficacy in SCLC treatment, which may be attributable to the abnormal vascular state in tumor microenvironment (TME).

In this report, we present a case of PD-1 inhibitors (ie, carrelizumab) combined with antiangiogenesis (ie, anlotinib) to treat advanced ES-SCLC.

## 2 Case presentation

**Figure 1:**
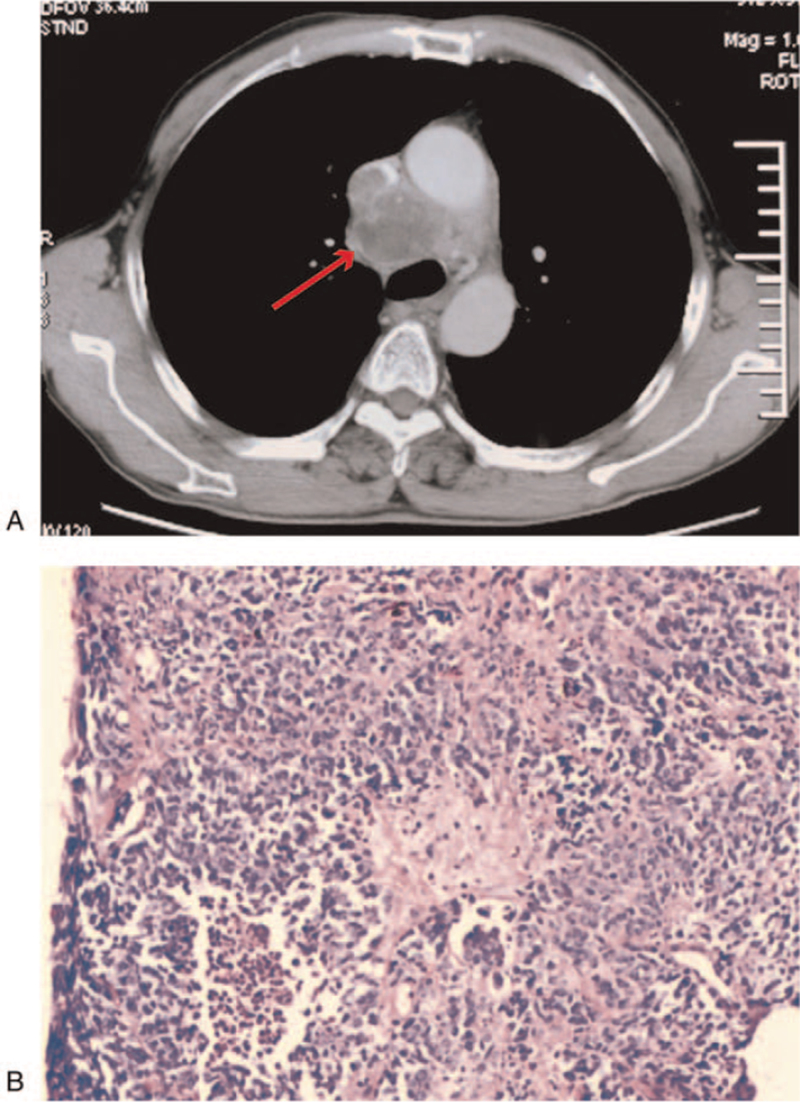
(A) Chest CT scan showed a mass in the right anterior upper mediastinum before treatment, January 16, 2019. (B) Histopathological findings of a biopsy specimen indicated SCLC (hematoxylin and eosin stain, magnification × 200). CT = computed tomography, SCLC = small cell lung cancer.

**Figure 2:**
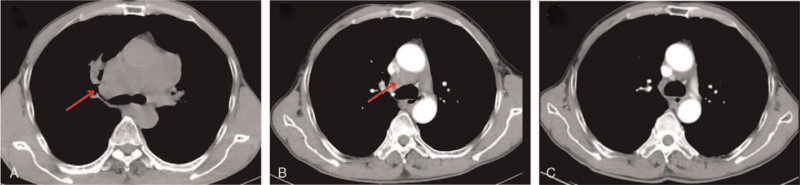
Chest CT of the patient after immunotherapy combined with chemotherapy/antiangiogenesis therapy. (A) Chest CT after camrelizumab combined with chemotherapy (June 19, 2019) showed the masses were larger than before. (B) The tumors significantly shrank after 4 cycles of camrelizumab combined with anlotinib (September 5, 2019). (C) The efficacy evaluation was CR (February 9, 2021). CR = complete remission, CT = computed tomography.

**Figure 3:**
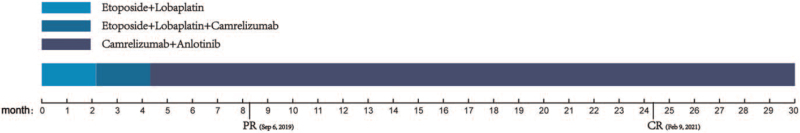
The treatment timeline of the patient. CR = complete remission, PR = partial response.

The current study includes a 55-year-old male patient, presenting cough and sputum for 1 month. Computed tomography (CT) scan showed that the right anterior upper mediastinum occupied space (Fig. 1A). There were multiple lymph nodes in the 2 clavicle areas, mediastinum, and 2 lung hilums, involving the superior vena cava and pericardial effusion. The head magnetic resonance showed no space-occupying, but the anterior edge of the left acetabulum showed abnormal signals. The disease was dignosed as SCLC by CT-guided needle biopsy of the mediastinal mass, and the tumor node metastasis staging was T2N3M1 (Stage IV, AJCC 8th Edition) ES-SCLC. In addition, pathological diagnosis also confirmed this conclusion (January 16, 2019, Fig. 1B). According to National Comprehensive Cancer Network, the patient received 4 cycles of first-line chemotherapy: etoposide (100 mg/m^2^, d1–3) combined with lobaplatin (30 mg/m^2^, d1) every 3 weeks for 4 cycles (January 25, 2019–March 29, 2019). After this treatment period, the CT examination evaluated that the mediastinal lesions were larger than before, evaluated as progressive disease according to the Response Evaluation Criteria in Solid Tumors guidelines. Subsequently, we improved the treatment. Based on the original plan, combined with camrelizumab (200 mg, d1, Q21d) for 2 cycles, re-examination of CT showed that the right clavicle area, mediastinum, 2 hilar nodules and masses were larger than before (Fig. 2A), evaluated as progressive disease. From June 21, 2019, the patient was treated with a PD-1 inhibitor and combined antiangiogenesis therapy; that is, intravenous infusion of camrelizumab (200 mg, d1, Q21d) plus oral anlotinib (12 mg, d1–14, Q21d). After 4 cycles, chest CT showed significant shrinkage in mediastinal lesions (Fig. 2 B), evaluation was a partial response. Encouraged by the initial result, we continued to use camrelizumab combined with anlotinib treatment. At the end of 26 cycles, the chest CT examination revealed that the patient had achieved complete remission (Fig. 2C). It was worth noting that the main side effect was hand–foot syndrome during the treatment period, and the adverse event was evaluated as graded 2 according to National Cancer Institute Common Toxicity Criteria for Adverse Events (NCI-CTC AE, version 4.0). To date, the PFS has been as long as 24 months since treated with camrelizumab and anlotinib. Considering this side effect may relate to the toxicity of anlotinib, we reduced the dosage to 10 mg, and found that the hand–foot syndrome reaction was reduced to grade 1. Figure 3 shows the treatment timeline of the patient.

The study was approved by the Hospital Ethics Committee of the Affiliated Lianyungang Hospital of Xuzhou Medical University and the patient provided written informed consent.

## 3 Discussion

SCLC is a differentiated neuroendocrine tumor with strong invasiveness, and most patients have developed to ES-SLCL at the time of initial diagnosis.^[1]^ The response rate among patients with SCLC to platinum-based combined chemotherapy can be as high as 75% to 95%, but the effective time is short, easy to relapse, and the median PFS is no more than 6 months.^[7]^ Therefore, several clinical studies of molecular targeting drugs have been conducted, such as targeted antiangiogenesis, cell signaling pathways, cell apoptosis, etc. Depressingly, those mentioned drugs did not show the potential to improve the prognosis of SCLC patients. With the development and application of ICIs, the treatment modalities of SCLC have been expanded and developed. In the IMpower133 study, atezolizumab combined with chemotherapy improved the survival benefit of ES-SCLC by 2 months.^[8]^ Moreover, durvalumab combined with chemotherapy increased survival by 2.7 months and reduced the risk of death by 27% in the CASPIAN study.^[9]^ Therefore, ICIs combination with chemotherapy is very likely to become a new first-line treatment for ES-SCLC.

The immune system plays an important role in oncogenesis and progression. Under normal physiological conditions, the immune system can recognize the “self” and avoid autoimmune reactions. Meanwhile, it can also recognize the “nonself” and resist foreign invasions appropriately. This is the type of “Art of Balance”. Through the expression of cell membrane antigens, cancer cells cleverly shield the recognition of immune cells and then escape from the immune system. Without protecting the immune system, tumor cells continue to proliferate, differentiate, and metastasize.^[10]^ As an inhibitory membrane receptor on T cells, PD-1 plays an important role in tumor immune evasion. Once binding with its ligand PD-L1, T cells activation will be inhibited. Based on the research results of CheckMate 032, KeyNote 028 and KeyNote158, Nivolumab, and Pembrolizumab as single drugs were approved for third-line treatment of ES-SCLC.^[11–13]^ However, the effect of the above PD-1 inhibitors on SCLC is limited, thus combined immunotherapies are becoming the main research direction. The KeyNote 604 study compared the efficacy of pembrolizumab and EP (etoposide and cis-platinum) combination with single EP treatments, the results suggested that the combined treatment improved median PFS (4.8 months vs 4.3 months, hazard ratio = 0.75, *P* = .0023), but did not improve overall median survival (10.8 months vs 9.7 months, hazard ratio = 0.80, *P* = .0164).^[14]^ Therefore, further improving the efficacy of PD-1 inhibitors for treating SCLC has become an urgent problem in clinical research.

Among the related factors that determine cancer immunity, TME has become the main obstacle affecting the efficacy of ICIs.^[15]^ In the abnormal physiological and biochemical environment of TME, the interaction between aberrant tumor blood vessels and immune cells severely interferes with the killing effect of immune cells on tumors and promotes tumor progression.^[16,17]^ First, the malignant proliferation of tumor cells makes the connections between reticulum cells and endothelial cells loose, lacks pericytes, and increases the permeability of blood vessels, leading to high interstitial fluid pressure around the tumor, and seriously hinders the flow of blood into the tumor tissue. In addition, reduced blood flow can cause tumor hypoxia, and tumor-specific cytotoxic T lymphocytes in plasma cannot penetrate TME and kill tumor cells.^[18,19]^ Besides, a chronic hypoxic environment can up-regulate the expression of PD-L1 by tumor cells and inactivate T cells in the tumor vessel lumen.^[20]^

Vascular endothelial growth factor (VEGF) also plays a key role in tumor angiogenesis. Previously we found that VEGF can bind to its receptor VEGFR2 and regulate hypoxia-inducible factor-1α through PI3K/Akt and MAPK/ERK signaling pathways, leading to tumor cell proliferation, antiapoptosis, and radioresistance.^[21]^ Kim et al^[22]^ found that VEGF up-regulates the expression of thymocyte selection-associated high mobility group box gene (TOX) in CD8^+^ T cells in TME, initiates the TOX-mediated transition to an immune-depleted state, and up-regulates multiple checkpoint inhibitory receptors on T cells. It is worth noting that VEGFR2 knocking out CD8^+^ T cells can down-regulate TOX expression and reactivate tumor-specific CD8^+^ T cells, confirming that targeting the VEGF/VEGFR2 pathway can reverse T cell depletion and enhance the antitumor effect of ICIs. Thus, VEGF of a tumor can affect the activity of immune cells in the tumor area through various mechanisms.

Camrelizumab is a PD-1 inhibitor, which can specifically bind to PD-1, thereby blocking the binding of PD-1/PD-L1 and the activation of downstream signaling pathways.^[23–24]^ Those positive results have been proved in several clinical studies.^[25]^ Anlotinib is a domestic small-molecule tyrosine kinase receptor inhibitor that can target VEGFR2 with strong selectivity, and has a promising target focusing effect. Anlotinib is recommended in the Chinese Society of Clinical Oncology guidelines as the third-line and above treatment for relapsed ES-SCLC. In this case, the tumor of patient continued to progress after chemotherapy and chemotherapy plus camrelizumab. In contrast, camrelizumab (PD-1 inhibitors) combined with anlotinib (antiangiogenic) therapy showed significant tumor shrinkage, and the clinical benefit was long-lasting. Therefore, this case proves to a certain extent that antiangiogenesis therapy can improve the efficacy of immunotherapy and prolong the time of clinical benefit.

## 4 Conclusion

We present a novel, effective combined immuno- and chemotherapy paradigm to treat ES-SCLC. Using PD-1 inhibitors (ie, carrelizumab) combined with antiangiogenesis (ie, anlotinib), the tumor of patient shrank significantly. This combined therapy may broaden the therapeutic avenue for patients with relapsed ES-SCLC. However, further large-scale clinical trials are required to prove its efficacy and safety.

## Author contributions

**Conceptualization:** Liang Liu.

**Data curation:** Liang Liu.

**Formal analysis:** Liang Liu.

**Investigation:** Liang Liu, Xin Zhang, Lihua Zhou, Tao Yang, Yun Qiao.

**Project administration:** Xiaodong Jiang.

**Writing – original draft:** Liang Liu.

**Writing – review & editing:** Xiaodong Jiang.

## References

[1] Deneka AY Boumber Y Beck T Golemis EA. Tumor-targeted drug conjugates as an emerging novel therapeutic approach in small cell lung cancer (SCLC). Cancers (Basel) 2019;11:1297. 10.3390/cancers11091297 PMC6769513 31484422

[2] Li D Xu X Liu J. Small cell lung cancer (SCLC) incidence and trends vary by gender, geography, age, and subcategory based on population and hospital cancer registries in Hebei, China (2008–2017). Thorac Cancer 2020;11:2087–93. 32589361 10.1111/1759-7714.13412 PMC7396395

[3] Calles A Aguado G Sandoval C Álvarez R. The role of immunotherapy in small cell lung cancer. Clin Transl Oncol 2019;21:961–76. 30637710 10.1007/s12094-018-02011-9

[4] Ando K Manabe R Kishino Y. Comparative efficacy and safety of immunotherapeutic regimens with PD-1/PD-L1 inhibitors for previously untreated extensive-stage small cell lung cancer: a systematic review and network meta-analysis. Curr Oncol 2021;28:1094–113. 33673470 10.3390/curroncol28020106 PMC8025754

[5] Melosky B Cheema PK Brade A. Prolonging survival: the role of immune checkpoint inhibitors in the treatment of extensive-stage small cell lung cancer. Oncologist 2020;25:981–92. 32860288 10.1634/theoncologist.2020-0193 PMC7648366

[6] Moya-Horno I Viteri S Karachaliou N Rosell R. Combination of immunotherapy with targeted therapies in advanced non-small cell lung cancer (NSCLC). Ther Adv Med Oncol 2018;10:1758834017745012. 29383034 10.1177/1758834017745012 PMC5784559

[7] Rossi A Di Maio M Chiodini P. Carboplatin- or cisplatin-based chemotherapy in first-line treatment of small-cell lung cancer: the COCIS meta-analysis of individual patient data. J Clin Oncol 2012;30:1692–8. 22473169 10.1200/JCO.2011.40.4905

[8] Horn L Mansfield AS Szczęsna A. First-Line atezolizumab plus chemotherapy in extensive-stage small-cell lung cancer. N Engl J Med 2018;379:2220–9. 30280641 10.1056/NEJMoa1809064

[9] Paz-Ares L Dvorkin M Chen Y. Durvalumab plus platinum-etoposide versus platinum-etoposide in first-line treatment of extensive-stage small-cell lung cancer (CASPIAN): a randomised, controlled, open-label, phase 3 trial. Lancet 2019;394:1929–39. 31590988 10.1016/S0140-6736(19)32222-6

[10] Seliger B. Strategies of tumor immune evasion. BioDrugs 2005;19:347–54. 16392887 10.2165/00063030-200519060-00002

[11] Antonia SJ López-Martin JA Bendell J. Nivolumab alone and nivolumab plus ipilimumab in recurrent small-cell lung cancer (CheckMate 032): a multicentre, open-label, phase 1/2 trial [published correction appears in Lancet Oncol. 2016 Jul;17(7):e270] [published correction appears in Lancet Oncol. 2019 Feb;20(2):e70]. Lancet Oncol 2016;17:883–95. 27269741 10.1016/S1470-2045(16)30098-5

[12] Ott PA Elez E Hiret S. Pembrolizumab in patients with extensive-stage small-cell lung cancer: results from the phase Ib KEYNOTE-028 study. J Clin Oncol 2017;35:3823–9. 28813164 10.1200/JCO.2017.72.5069

[13] Chung HC Piha-Paul SA Lopez-Martin J. Pembrolizumab after two or more lines of previous therapy in patients with recurrent or metastatic SCLC: results from the KEYNOTE-028 and KEYNOTE-158 studies. J Thorac Oncol 2020;15:618–27. 31870883 10.1016/j.jtho.2019.12.109

[14] Rudin CM Awad MM Navarro A. Pembrolizumab or placebo plus etoposide and platinum as first-line therapy for extensive-stage small-cell lung cancer: randomized, double-blind, phase III KEYNOTE-604 Study. J Clin Oncol 2020;38:2369–79. 32468956 10.1200/JCO.20.00793 PMC7474472

[15] Yi M Jiao D Qin S Chu Q Wu K Li A. Synergistic effect of immune checkpoint blockade and anti-angiogenesis in cancer treatment. Mol Cancer 2019;18:60. 30925919 10.1186/s12943-019-0974-6 PMC6441150

[16] De Palma M Biziato D Petrova TV. Microenvironmental regulation of tumour angiogenesis. Nat Rev Cancer 2017;17:457–74. 28706266 10.1038/nrc.2017.51

[17] Xia A Zhang Y Xu J Yin T Lu XJ. T cell dysfunction in cancer immunity and immunotherapy. Front Immunol 2019;10:1719. 31379886 10.3389/fimmu.2019.01719 PMC6659036

[18] Baluk P Hashizume H McDonald DM. Cellular abnormalities of blood vessels as targets in cancer. Curr Opin Genet Dev 2005;15:102–11. 15661540 10.1016/j.gde.2004.12.005

[19] Lugano R Ramachandran M Dimberg A. Tumor angiogenesis: causes, consequences, challenges and opportunities. Cell Mol Life Sci 2020;77:1745–70. 31690961 10.1007/s00018-019-03351-7 PMC7190605

[20] Motz GT Santoro SP Wang LP. Tumor endothelium FasL establishes a selective immune barrier promoting tolerance in tumors. Nat Med 2014;20:607–15. 24793239 10.1038/nm.3541 PMC4060245

[21] Liu L Qiao Y Hu C. Endostatin exerts radiosensitizing effect in non-small cell lung cancer cells by inhibiting VEGFR2 expression. Clin Transl Oncol 2016;18:18–26. 26542176 10.1007/s12094-015-1319-6

[22] Kim CG Jang M Kim Y. VEGF-A drives TOX-dependent T cell exhaustion in anti-PD-1-resistant microsatellite stable colorectal cancers. Sci Immunol 2019;4:eaay0555. 31704735 10.1126/sciimmunol.aay0555

[23] Zhou C Chen G Huang Y. Camrelizumab plus carboplatin and pemetrexed versus chemotherapy alone in chemotherapy-naive patients with advanced non-squamous non-small-cell lung cancer (CameL): a randomised, open-label, multicentre, phase 3 trial. Lancet Respir Med 2021;9:305–14. 33347829 10.1016/S2213-2600(20)30365-9

[24] Fan Y Zhao J Wang Q. Camrelizumab plus apatinib in extensive-stage SCLC (PASSION): a multicenter, two-stage, phase 2 trial. J Thorac Oncol 2021;16:299–309. 33166719 10.1016/j.jtho.2020.10.002

[25] Yang S Zhang Z Wang Q. Emerging therapies for small cell lung cancer. J Hematol Oncol 2019;12:47. 31046803 10.1186/s13045-019-0736-3 PMC6498593

